# The bovine cumulus proteome is influenced by maturation condition and maturational competence of the oocyte

**DOI:** 10.1038/s41598-020-66822-z

**Published:** 2020-06-18

**Authors:** J. Walter, C. Monthoux, C. Fortes, J. Grossmann, B. Roschitzki, T. Meili, B. Riond, R. Hofmann-Lehmann, H. Naegeli, U. Bleul

**Affiliations:** 1 Clinic of Reproductive Medicine, Vetsuisse Faculty, University of Zurich, Zurich, Switzerland; 2 Functional Genomics Centre Zurich, University and ETH Zurich, Zurich, Switzerland; 3 Clinical Laboratory, Department of Clinical Diagnostics and Services, Vetsuisse Faculty, University of Zurich, Zurich, Switzerland; 4 Institute of Pharmacology and Toxicology, Vetsuisse Faculty, University of Zurich, Zurich, Switzerland; 5 Center for Clinical Studies, Vetsuisse Faculty, University of Zurich, Zurich, Switzerland; 6 Swiss Institute of Bioinformatics, (SIB), Zurich, Switzerland

**Keywords:** Prognostic markers, Preclinical research, Animal biotechnology, Proteomics

## Abstract

*In vitro* maturation (IVM) of oocytes has still a negative impact on the developmental competence of oocytes. Therefore, this study analysed the cumulus proteome of individual cumulus-oocyte complexes (COCs) with and without maturational competence, matured under *in vivo* or *in vitro* conditions (n = 5 per group). A novel, ultrasensitive mass spectrometry (MS) based protein profiling approach, using label-free quantification, was applied. The detected cumulus proteome included 2226 quantifiable proteins and was highly influenced by the maturation condition (479 differentially expressed proteins) as well as maturational competence of the corresponding oocyte (424 differentially expressed proteins). Enrichment analysis showed an overrepresentation of the complement and coagulation cascades (CCC), ECM-receptor interaction and steroid biosynthesis in cumulus of COCs that matured successfully under *in vivo* conditions. Verification of the origin of CCC proteins was achieved through detection of C3 secretion into the maturation medium, with significantly increasing concentrations from 12 (48.4 ng/ml) to 24 hours (68 ng/ml: p < 0.001). In relation, concentrations in follicular fluid, reflecting the *in vivo* situation, were >100x higher. In summary, this study identified important pathways that are impaired in IVM cumulus, as well as potential markers of the maturational competence of oocytes.

## Introduction

Maturation is the final step of oogenesis where oocytes undergo meiotic resumption to prepare for fertilization. For the *in vitro* production of embryos, this crucial step can still take place *in vivo* through hormonal stimulation of the oocyte donor. These oocytes possess typically a higher developmental potential compared to their *in vitro* matured counterparts^1^. Therefore, for healthy women, ovarian stimulation using gonadotrophins is still a common part of the IVP procedure for infertility treatment^2^, even though it can result in an excessive response causing the ovarian hyperstimulation syndrome^3^. In cattle, the *in vitro* production procedure is usually performed using *in vitro* maturation of oocytes, accepting impaired developmental rates. Despite high maturation rates of up to 90% of immature bovine oocytes under *in vitro* conditions, usually not more than 40% develop until the blastocyst stage^4–8^. This is a substantially lower rate compared to the *in vitro* production of embryos from *in vivo* matured oocytes, for which a blastocyst rate of 73% was recorded^9^. Also, the expansion rate of blastocysts was reduced after maturation *in vitro* (12%) compared to maturation *in vivo* (41%)^10^. Early studies on oviductal transfer of *in vitro* or *in vivo* matured oocytes before insemination^11^ or just after *in vitro* fertilization^12^ showed similar effects. Increased rates of developmental abnormalities were described for *in vitro* produced embryos^13^. This might especially be the result of maturation *in vitro*, which results in a higher degree of chromosomal abnormalities and decreased cell counts per blastocyst^10^. All these findings suggest that the *in vivo* situation is not sufficiently reflected in *in vitro* maturation systems. Media used for the *in vitro* maturation (IVM) were initially developed for somatic cells culture and underwent empirical adaptations^14,15^. These culture conditions are very static, providing the same microenvironment for the whole maturation period^16^. Adjustment of protocols towards more physiological conditions is highly desirable to improve the developmental competence of *in vitro* matured oocytes.

During maturation, a close bidirectional exchange of metabolites takes place between oocytes and their accompanying somatic cells of the cumulus complex (CC). The CC facilitates the favourable microenvironment necessary for oocyte growth and development through transfer of metabolic substrates, elimination of toxic metabolites and modulation of environmental influences. The presence of cumulus cells during IVM is directly linked with an improved developmental potential of the oocyte^17,18^. As a consequence, understanding aberrations of metabolism *in vitro* in the cells with the most intimate contact to the oocyte provides highly valuable information for a closer mimicking of the natural maturation environment.

The influence of *in vitro* maturation on gene expression in cumulus cells and oocytes was evaluated in several previous studies^9,19–23^. Data on the altered cumulus-oocyte complex (COC) proteome after maturation *in vitro* are only scarce. Global proteomic studies on COCs were conducted on pooled samples, comparing oocytes with cumulus cells^24,25^ or analysing the influence of maternal age^26^. Up to now, sample pooling was necessary as a result of the limited amount of available COC material for protein analysis – where no enrichment steps are available as for gene expression analysis. Nowadays, technical advances provide a more sensitive detection of low-abundance proteins, which allows for the analysis at a single oocyte level^27–29^. Pooling of samples has the benefit to reduce the analysis time, but estimation of inter-individual differences is not possible. This reduces the applicability of the approach for biomarker discovery^30,31^. Especially the opportunity to relate the cumulus proteome to the maturational and developmental competence of the corresponding oocyte can contribute to the discovery of novel biomarkers for oocyte selection in assisted reproduction^32,33^.

In this study, cumulus proteomes after maturation *in vivo* and *in vitro* were compared with each other. The results will contribute to a better understanding of the limitations for maturation *ex vivo*. Beyond this, correlation of the cumulus proteome to the maturation stage of the corresponding oocyte might reveal potential biomarkers to predict the oocytes maturational competence.

## Results

### Proteome analysis

**Figure 1:**
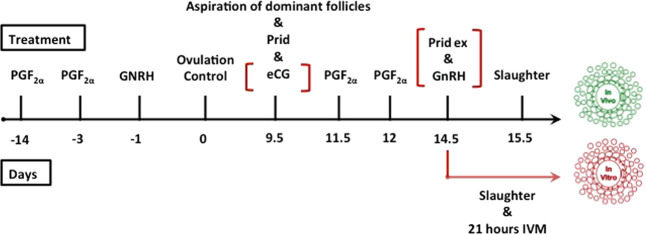
Simplified scheme of synchronisation and superovulation treatments for the collection of cumulus-oocyte complexes (COCs) after *in vivo* and *in vitro* maturation. The applications in red brackets were performed only on animals slaughtered on day 14.5 and the COCs maturated *in vitro* (PGF2α: prostaglandin FF2α; eCG: equine chorionic gonadotrophin; Prid: progesterone-releasing intravaginal device; GnRH: gonadotrophin releasing hormone).

**Figure 2:**
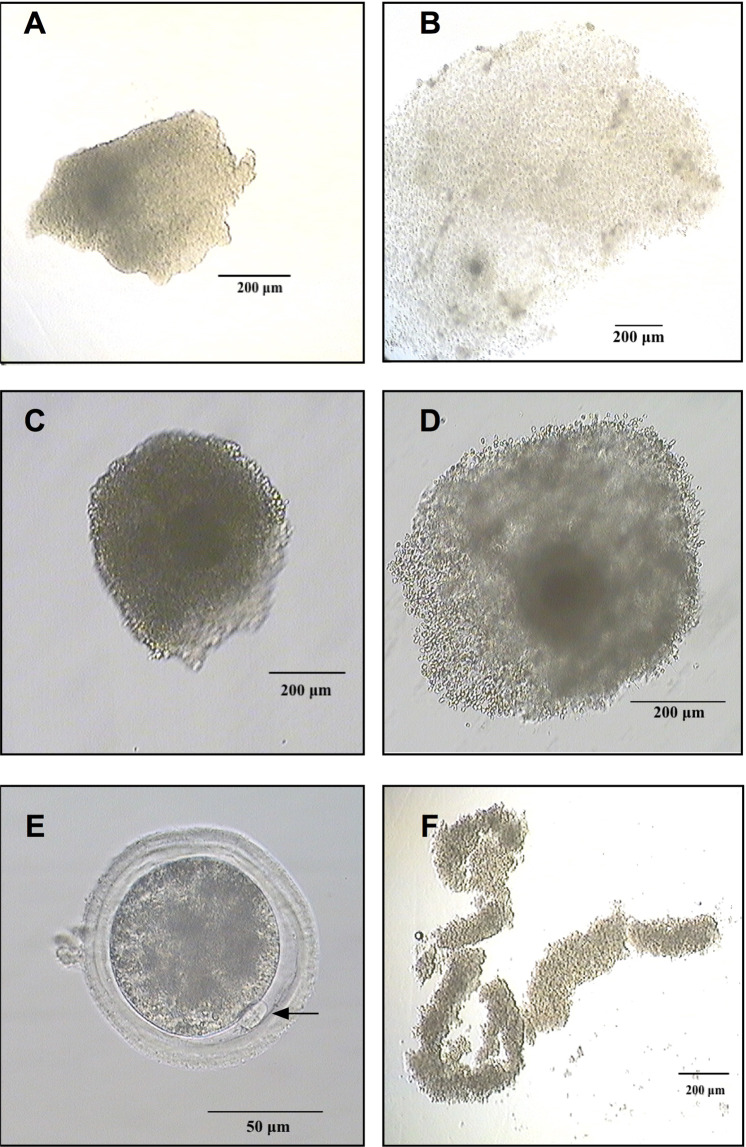
COC that failed to mature *in vivo* (**A**); COC successfully matured *in vivo* (**B**); COC that failed to mature *in vitro* (**C**); COC successfully matured *in vitro* (**D**); *in vitro* successfully matured oocyte with extruded first polar body (**E**); cumulus sample collected for proteomic analysis of a COC that failed to mature *in vivo* (**F**).

The study compared the cumulus proteome of oocytes with and without maturational competence that were matured either under *in vivo* or *in vitro* conditions. In total, twenty cumulus samples corresponding to single oocytes were examined, using a mass spectrometry (MS) protein profiling approach. The two maturation conditions included five cumulus samples from oocytes that matured successfully (extrusion of 1^st^ polar body) or failed to mature. Donors of cumulus-oocytes complexes (COCs) matured under *in vivo* conditions were oestrous synchronised, eCG superovulated and slaughtered 24 hours after final GnRH injection and progesterone withdrawal. COCs for the *in vitro* group were collected after oestrous synchronisation on day 5 of progesterone treatment and matured in single culture for 21 hours (Fig. 1). Cumulus samples were removed from their oocyte, washed in PBS, snap frozen and stored in liquid nitrogen. Extrusion of first polar body was assessed in fully denuded oocytes to confirm the maturation success and classify the COCs in successfully matured or failed to mature (Table 1; Fig. 2). Protein profiling of the cumulus samples was conducted in a label-free MS approach. For cell lysis and protein digestion, an adapted filter-aided sample preparation protocol was used. Data were analysed by label-free quantification using ProgenesisQI software (NonlinearDynamics).

**Table 1:** Overview on COCs collected from the six heifers.

Heifer	*in vivo*			Heifer	*in vitro*		
	1	2	3		4	5	6
Total COCs collected (successfully matured)	44 (9)	4 (2)	27 (20)	Total COCs in maturation (successfully matured)	9 (6)	13 (5)	6 (4)
	COCs in final analysis				COCs in final analysis		
Successfully Matured	4	1	0	Successfully Matured	3	1	1
Failed to Mature	4	0	1	Failed to Mature	1	3	1

COCs with a sufficient amount of cumulus were selected for final analysis.

**Figure 3:**
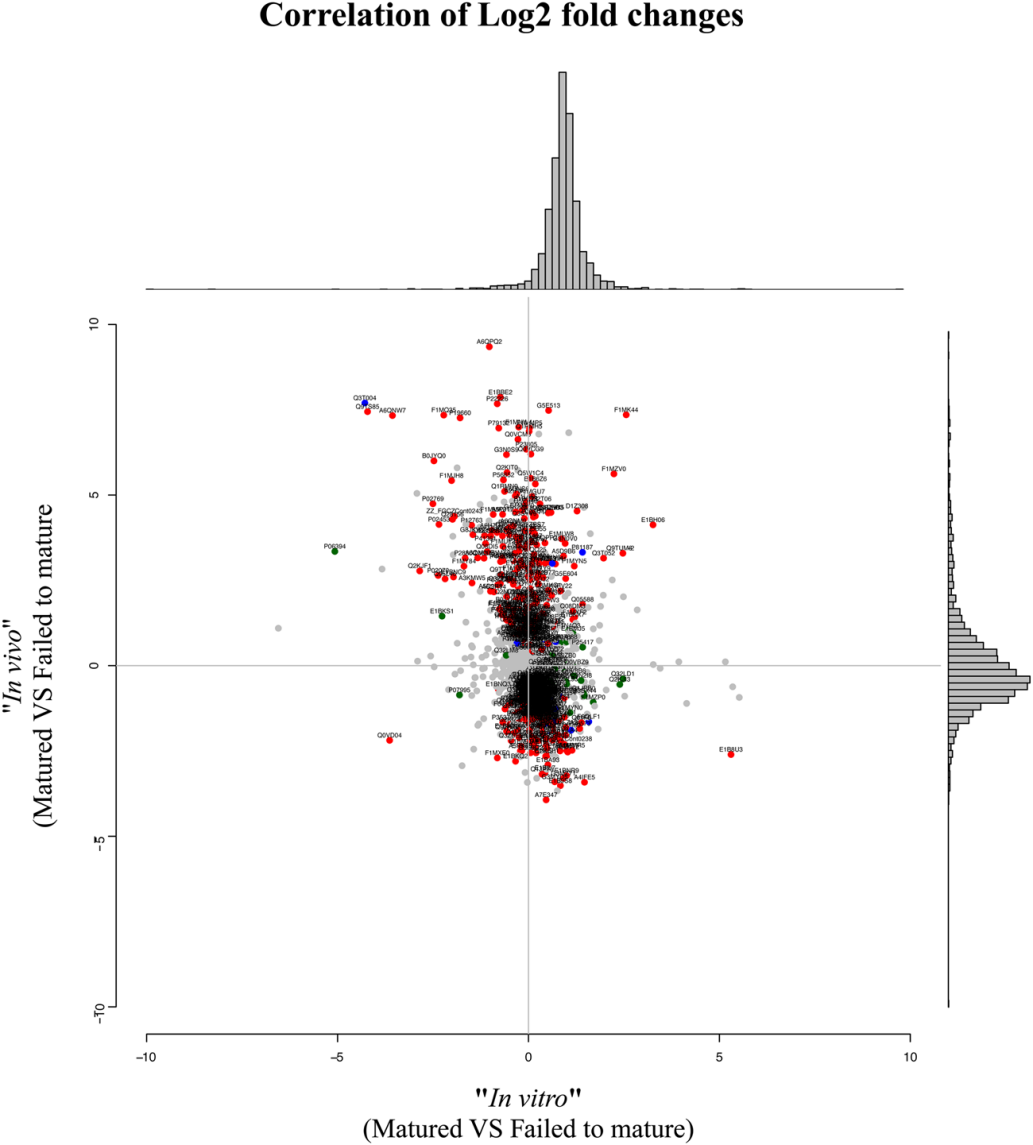
Plotted are the log2 fold-changes between *in vitro* (x-axis) and *in vivo* (y-axis) sucessfully matured versus failed to mature instances along with a histogram on the top panel and the right panel where the spread of the changes is visualized. Colored and labeled proteins are those where the p-value from the respective two-group comparison is significant with respect to 0.05 (green = *in vitro*, red = *in vivo*, blue = both).

In the 20 examined cumulus samples, a total of 2226 proteins were quantifiable (with ≥ 2 peptides per protein, estimated protFDR <1%). The four biological groups underwent pairwise comparisons to identify statistically relevant changes in protein abundance. For significant differences, a fold change in protein expression >2 along with p < 0.05 (t-test) was considered. A greater biological heterogeneity was observed in the *in vivo* matured samples compared to their *in vitro* matured counterparts (Fig. 3). In the *in vitro* matured group, a majority of proteins was up-regulated in CC samples of COCs that failed to mature. The highest fold changes were achieved in CC matured successfully under *in vivo* conditions.

The four different two group analyses revealed the following statistically significant results:

#### Comparison group “Matured”

In cumulus samples from successfully matured COCs, 479 proteins were significantly differentially expressed between *in vitro* and *in vivo* maturation (see Supplemental Data Table 1 for the complete list of proteins). 235 proteins were up-regulated in the *in vivo* group and 244 were up-regulated in the *in vitro* group.

#### Comparison group *“In vivo”*

In cumulus samples of COCs that underwent *in vivo* maturation, 424 proteins were significantly differentially expressed between the two maturation outcomes (see Supplemental Data Table 2 for complete lists). 222 of these proteins were up-regulated in the COCs that matured successfully *in vivo* and the other 202 in the group that failed to mature *in vivo*.

#### Comparison group “Failed to mature”

In the cumulus samples from the COCs that failed to mature under both maturation conditions, 175 proteins were significantly differentially expressed after *in vitro* and *in vivo* maturation (see Supplemental Data Table 3 for complete lists). 79 proteins were up-regulated in the COCs that failed to mature *in vivo* and 96 were up-regulated in the group that failed to mature *in vitro*.

#### Comparison group *“In vitro”*

In the cumulus collected after *in vitro* maturation of the COCs, only 20 proteins were significantly differentially expressed between cumulus that matured successfully failed to mature *in vitro* (see Supplemental Data Table 4 for complete lists). Most up-regulated proteins (16) were in the group that matured successfully, compared to the COCs that failed to mature (4).

**Figure 4:**
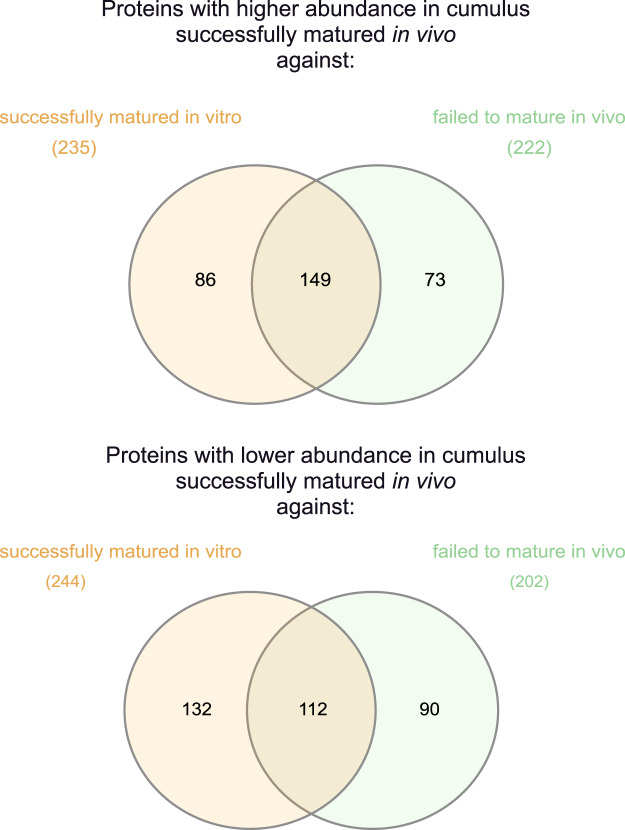
Venn diagrams for the comparison of significantly different results (fold change >2 along with p ≤ 0.05) between the “successfully matured *in vivo”* group against the groups “failed to mature *in vivo*” and “successfully matured *in vitro”*. The upper diagram shows the proteins with higher abundance, the lower diagram the proteins with lower abundance in the “successfully matured *in vivo*” group. The left side (orange) shows the comparison against the “successfully matured *in vitro*” group, the right side (green) shows the comparison against the “failed to mature *in vivo*” group. In total, 381 proteins with significantly different expression were not shared between these two group comparisons, whereas 261 were shared.

Comparison of the groups “Matured” and “*In vitro*”, revealed 261 shared proteins among the significantly differentially expressed proteins (Fig. 4, Supplemental Data Table 5). Of these, 149 proteins were up- and 112 down-regulated in *in vivo* successfully matured cumulus. In total, 381 proteins with significantly different expression were not shared between these two group comparisons.

**Figure 5:**
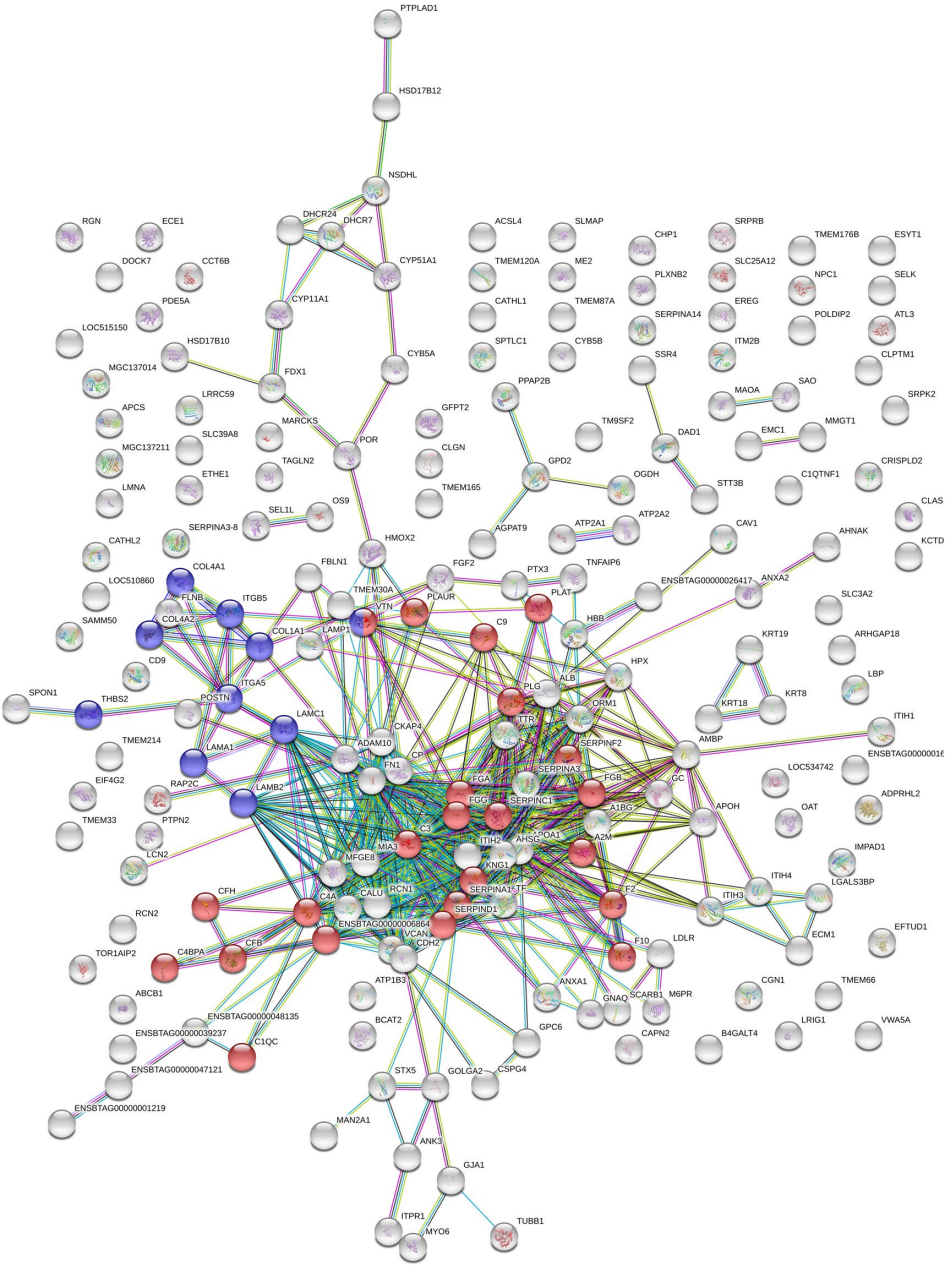
STRING-DB interaction network of proteins overexpressed in *in vivo* successfully matured cumulus compared to *in vivo* failed to mature cumulus (minimum required interaction score: 0.7). Of the 222 significantly different expressed proteins 200 matched the database. Significantly overrepresented were the KEGG pathway complement and coagulation cascades (n = 23; red) and ECM receptor interaction (n = 10, blue).

**Figure 6:**
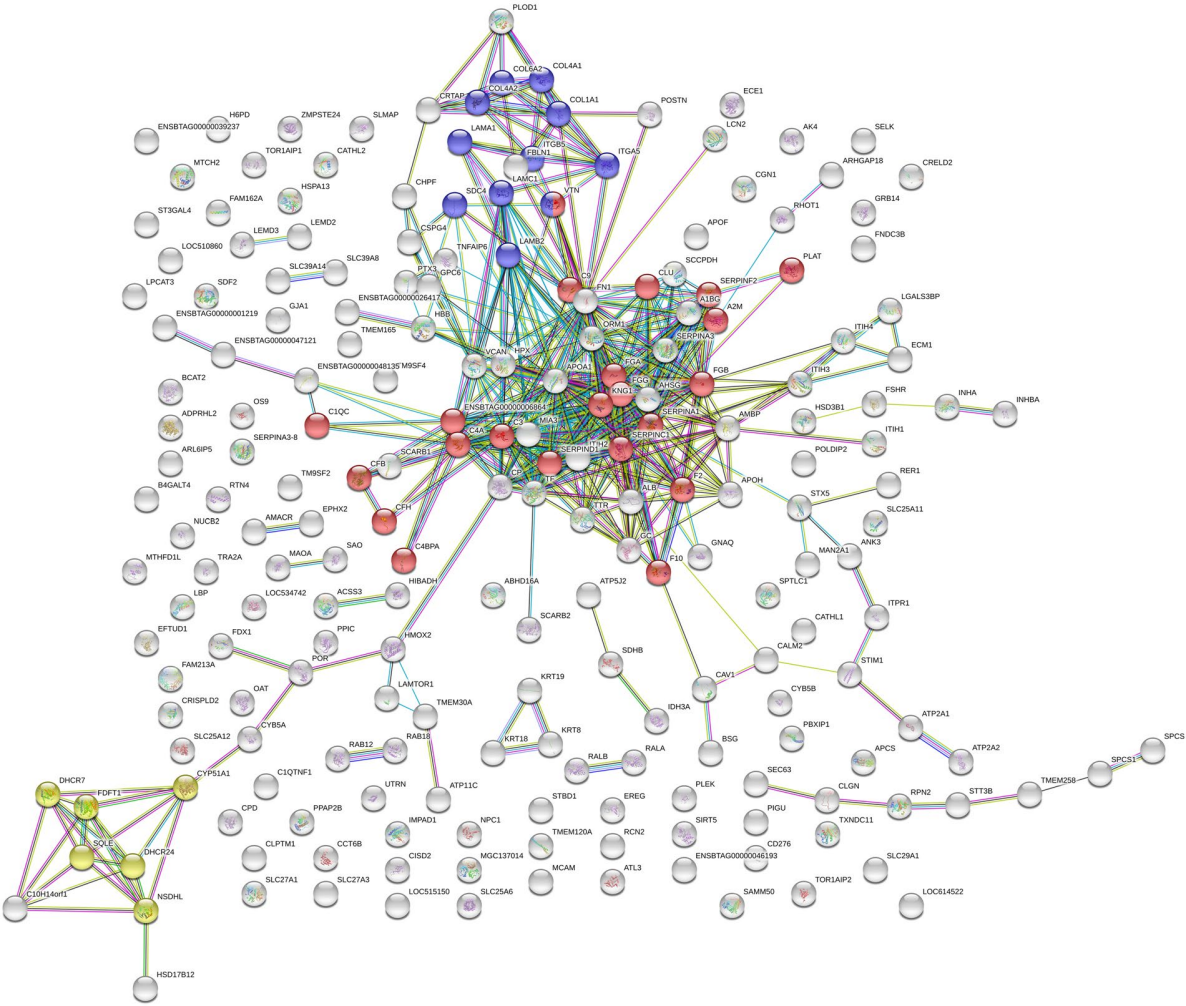
STRING-DB interaction network of proteins overexpressed in *in vivo* successfully matured cumulus compared to *in vitro* successfully matured cumulus (minimum required interaction score: 0.7). Of the 235 significantly different expressed proteins 215 matched the database. Significantly overrepresented were the KEGG pathways complement and coagulation cascades (n = 22; red), ECM receptor interaction (n = 11, blue) and steroid biosynthesis (n = 6, yellow).

An enrichment analysis using StringDB software (www.string-db.org) revealed significantly overrepresented KEGG (Kyoto Encyclopedia of Genes and Genomes) pathways amongst proteins up-regulated in *in vivo* successfully matured COCs (Figs. 5 and 6, Supplemental Data Table 6). The predominant finding was the overrepresentation of the KEGG pathway complement and coagulation cascade (CCC; map04610) in *in vivo* successfully matured cumulus samples. This was significant against the *in vivo* failed to mature as well as the *in vitro* successfully matured group (Figs. 5 and 6). Additionally, canonical pathway analysis was performed using ingenuity pathway analysis (IPA). Table 2 illustrates the comparison of the results for the most important two group analysis (*In vivo*: successfully matured against failed to mature; Matured: *in vivo* against *in vitro*).

**Table 2:** Comparison of canonical pathway analysis for the group comparisons “*In vivo*’’ and “Matured’’ based on log2 fold change (used cut offs: FC > 2; p < 0.05) using ingenuity pathway analysis (IPA).

Canonical Pathway	“*In vivo*” (Matured VS Failed to mature)	“Matured” (*In vivo* VS *In vitro*)
LXR/RXR Activation	4.26	4.38
Intrinsic Prothrombin Activation Pathway	1.89	1.41
Production of Nitric Oxide and Reactive Oxygen Species in Macrophages	0.82	2.33
Acute Phase Response Signaling	1.79	1.34
IL-8 Signaling	−2.12	−0.45
Nitric Oxide Signaling in the Cardiovascular System	−1.41	−1.13
Neuregulin Signaling	−1.34	−1.00
PDGF Signaling	−1.34	−1.00
PAK Signaling	−1.34	−1.00
Calcium-induced T Lymphocyte Apoptosis	2.24	N/A
Non-Small Cell Lung Cancer Signaling	−1.00	−1.00
STAT3 Pathway	−2.00	N/A
Integrin Signaling	−0.58	1.41
NRF2-mediated Oxidative Stress Response	0.00	−1.89
PPARÎ ± /RXRÎ ± Activation	0.71	1.13
ILK Signaling	0.71	1.13
GNRH Signaling	−0.82	−0.45
Glioma Invasiveness Signaling	0.82	0.45
p70S6K Signaling	−0.82	−0.45
PTEN Signaling	0.82	0.45
Cholecystokinin/Gastrin-mediated Signaling	−1.13	0.00
Thrombin Signaling	−0.71	0.38
Agrin Interactions at Neuromuscular Junction	−1.00	N/A
EGF Signaling	−1.00	N/A
Coagulation System	0.53	−0.28
Ephrin Receptor Signaling	−0.33	0.45
IL-6 Signaling	0.45	0.00
Huntington’s Disease Signaling	0.45	0.00
G Beta Gamma Signaling	−0.45	0.00
Regulation of Cellular Mechanics by Calpain Protease	−0.38	0.00
Osteoarthritis Pathway	0.00	0.38

### Complement C3 ELISA

**Figure 7:**
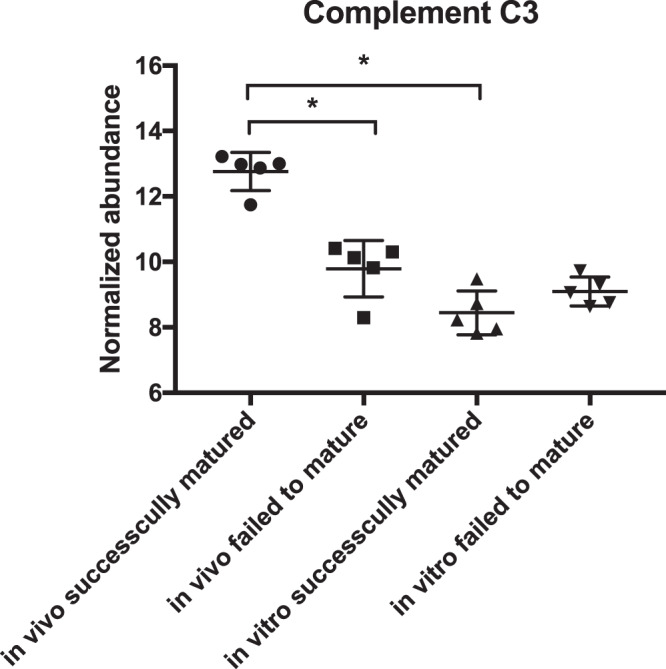
Normalized abundance (Progenesis QI; hyperbolic arcsine transformed) of complement C3 in the four analysed groups. The asterisk indicates statistically significant differences.

**Figure 8:**
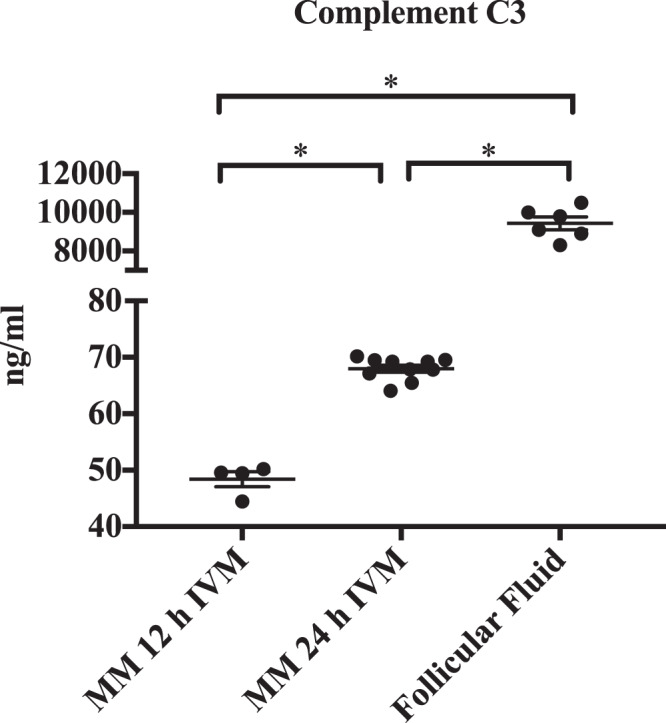
Measurement (ELISA) of Complement C3 concentration in maturation medium after 12 hours of IVM (mean and SEM). (n = 4; 48.4 ± 1.3 ng/ml), 24 hours of IVM (n = 10; 68 ± 0.6 ng/ml) as well as in follicular fluid of dominant follicles (n = 6; 9433 ± 330 ng/ml). Concentrations were significantly different (*; Mann Whitney Test: p ≤ 0.001).

The presence of the complement system in follicular fluid was already described in the literature^34–36^, but the origin was not elucidated up to now. Human granulosa cells express several genes of the complement system^37^. To verify the origin of the complement proteins in the cumulus samples of this study (Fig. 7), complement component 3 (C3), a central player in the complement and coagulation cascade, was analysed in maturation medium using a commercially available ELISA kit. In all control medium samples without COC contact C3 was not detectable. Mean C3 concentration in medium containing pools of 35 COCs was 48.4 ng/ml after 12 h and 68 ng/ml after 24 h. To bring these concentrations in relation to the natural conditions, analysis of follicular fluid (n = 6) was performed, where concentrations >100x higher than in the analysed maturation media were detected (mean concentration: 9433 ng/ml; Fig. 8).

## Discussion

This study used a novel highly sensitive approach to characterize the proteome of single bovine CCs after maturation *in vivo* or *in vitro*^29,38^. This proteome included 2226 proteins, of which a high percentage of significantly different proteins were detected (up to 21%). Similar protein detection was only achieved in previous studies with pooled CCs of several hundred COCs^24,25^. In the future, our new method will offer the opportunity to relate the cumulus proteome directly to the developmental potential of the corresponding oocyte, as already performed on gene expression level^33,39^.

More proteins were significantly up-regulated after *in vivo* than *in vitro* maturation (Fig. 3). Previous studies on bovine cumulus cells at gene expression level observed a similar upregulation for *in vivo* matured COCs^20^. A global protein expression profile in cumulus complexes from different maturation conditions and outcomes has not been reported so far. The *in vivo* condition in this study is represented by COCs collected after super-stimulation of donor animals. This promotes the growth of a majority of follicles that would otherwise undergo atresia^40^. Cumulus cell gene expression showed significant differences after superovulation of cows^41,42^. Therefore, the results will not be fully transferable to cumulus of naturally *in vivo* matured dominant follicles. Especially eCG, which was used for super-stimulation in this study, influences the transcriptional profile of COCs. Particularly affected are genes involved in lipid metabolism and oxidative stress^43^.

Interestingly, the differentially expressed proteome of cumulus from COCs successfully matured *in vitro* shows similarities with the failed to mature *in vivo* group (Fig. 4; 261 shared differentially expressed proteins). This is also reflected in the canonical pathway analysis (Table 2), where a similar enrichment and depletion of pathways between these two group comparisons was observed. Still, Fig. 4 (and Supplemental Data Table 5) also illustrates big differences in the proteome of these two groups, with a total of 381 proteins that were only significant in one of the two comparisons.

Pathways overrepresented in this proteomic study were complement and coagulation cascade, steroid biosynthesis and ECM-formation after maturation *in vivo* (Figs. 5 and 6, Supplemental Data Table 6). Interestingly, these pathways were underrepresented at gene expression level in a similar designed study^9^. The overexpression of the complement and coagulation pathway in *in vivo* matured cumulus compared to the *in vitro* matured counterparts was already detected for equine COCs^29^. In this bovine study, the KEGG pathway complement and coagulation cascade was overrepresented in *in vivo* successfully matured cumulus against *in vitro* successfully matured as well as *in vivo* failed to mature cumuli (Supplemental Data Table 6). The up-regulated complement proteins are involved in all three activation pathways of the complement cascade: the classical, the alternative and the lectin pathway. All these pathways activate the central C3 component to trigger the inflammatory process^34^. Proteins of the complement system are widely expressed in the female reproductive tract and were already detected in cumulus cells and oocytes^36,44,45^. The complement system was already characterized as an important constituent of the follicular fluid^34,35,37,46^. RNA analysis of granulosa cells revealed that complement factors are actively produced by these somatic cells^37^. The accumulation of C3 in maturation medium in this study supports this hypothesis. Nevertheless, concentrations in maturation medium were only a fraction of the concentration measured in follicular fluid (Fig. 8). Thus, underrepresentation of the complement system, especially C3, in the *in vitro* surrounding of the COC, might play a role in the reduced maturational competence of the oocyte. A favouring role of complement proteins in the preovulatory environment of the oocyte on its further developmental potential was already reported^34,47,48^. This hypothesis was supported by the positive effect on the oocytes developmental potential achieved by supplementation of bovine follicular fluid to IVM media^49^. The central complement component 3 (C3), up-regulated in the present study in *in vivo* successfully matured cumulus, seems to play a role in the fertilization process^50,51^. In the pig, a cleavage product of C3 (iC3b) has a positive influence on the maturation outcome^36^. This might explain the overexpression of the complement cascade in CCs that matured successfully in this study. The complement cascade overlaps with the coagulation cascade, which also plays an important role in the follicular fluid^52,53^. An influence of proteins from the coagulation system in the follicular fluid on *in vitro* fertilization outcome for the human species has already been detected before^54^. The coagulation system plays also a role in the ovulation process. It influences oocyte delivery to the oviduct by modulation of follicular fluid consistency and its impact on inflammatory cells^54,55^. The central component of the coagulation system - fibrinogen - is overexpressed after maturation *in vivo*. Fibrinogen gene expression in cumulus cells is up-regulated 6 h after the LH surge in cows compared to 2 h before the LH surge. These results give reason to assume that fibrinogen might play a role in triggering the final maturation^56^. The lack of complement proteins in *in vitro* matured CCs and in CCs that failed to mature under *in vivo* conditions strengthens the hypothesis of an important role of the coagulation cascade in triggering the full maturational potential of the COC.

Proteins of the ECM-receptor interaction pathway (map04512) were also overexpressed in *in vivo* successfully matured cumulus compared to *in vitro* successfully matured and *in vivo* failed to mature samples (Supplemental Data Table 6; Figs. 5 and 6). During maturation, expression of these proteins builds up cumulus extracellular matrix (ECM) responsible for interaction of the matrix with the surrounding environment. Collagens, laminins, fibronectin and vitronectin are all ligands of the transmembrane receptor integrin. The overexpression of these proteins in the present *in vivo* successfully matured COCs correlates with previous studies. Collagens, laminins and integrins showed increased expression during cumulus mucification in the cow^57^. Vitronectin and fibronectin were already detected in bovine cumulus cells and in the cumulus ECM matrix during maturation, with an increased expression after maturation^58,59^.

ECM-receptor interaction proteins play a role in post-maturation events: they are possibly involved in the maintenance of the expanded matrix around cumulus cells and the oocyte for oviductal pick-up, they influence sperm motility and are involved in gamete adhesion and fertilization^60–64^. Of the ECM proteins, vitronectin seems to play a special role for sperm-oocyte interactions with dose dependent effects^59,65^. Increased vitronectin in extracellular matrix showed negative effects on sperm motility and egg-sperm interactions^65^. Beside the reduced fertilization and sperm penetration rate, a reduction of polyspermy in cumulus-enclosed oocytes could be observed with high vitronectin concentrations in medium^65^. The increased expression of vitronectin in *in vivo* matured cumulus samples might play a role in blocking polyspermy in bovine *in vivo* matured COCs. Under *in vitro* conditions, an increased risk for polyspermy in cattle was described^66^. This link might be explained by the current findings, where a lack of proteins involved in ECM-receptor interaction in successfully *in vitro* matured COCs was detected.

Finally, the KEGG pathway steroid biosynthesis (map00100) was overrepresented in *in vivo* successfully matured compared to *in vitro* successfully matured cumulus (Supplemental Data Table 6, Fig. 6). These overexpressed proteins are all active in cholesterol biosynthesis. Similar results were found on gene expression level. Genes involved in cholesterol biosynthesis are down-regulated in human cumulus after IVM compared to maturation *in vivo*^23^. This effect may be influenced by the super-stimulation of the donors for *in vivo* matured COCs. Super-stimulatory protocols modify the steroidogenic capacity of bovine granulosa cells, which resulted in increased cholesterol concentrations in follicular fluid, as well as higher plasma estradiol concentrations^67^. The overrepresentation of this KEGG pathway was strengthened by the Ingenuity Pathway Analysis (Table 2), with LXR/RXR activation as the dominating canonical effect. The role of this system in the COC is not well characterized up to now. Still, LXR/RXR activation was already identified as a player in human granulosa cells as well as in follicular fluid^37,68^. The system contributes to cholesterol homeostasis and lipid metabolism in many tissues^69^. Cholesterol is an important compound with a role as membrane component or precursor in steroid hormone production and lipid metabolism. Cumulus cells produce cholesterol for the oocyte where it is required for the final nuclear maturation and progesterone production^70,71^. Further functions like oxidative stress defence were also attributed to proteins involved in cholesterol biosynthesis^72^. Especially the altered cumulus cell lipid metabolism after IVM is already known^22^. The underrepresentation of these pathways involved in cholesterol biosynthesis and homeostasis under *in vitro* maturation conditions indicates that the COC matured under non-physiological conditions, even when successfully matured, suffer from reduced local cholesterol synthesis. This can cause a variety of detrimental effects resulting in reduced developmental capacity of the corresponding oocyte.

## Conclusion

In the present study, characterization of the bovine cumulus proteome was conducted, for the first time, at the single COC level using an ultrasensitive LC-MS/MS approach. The proteome of the cumulus was highly influenced by the maturation condition as well as the maturational competence of the corresponding oocyte. Under *in vivo* conditions, successfully matured COCs showed strong influence of KEGG pathways of the complement and coagulation cascade, ECM-receptor interactions and steroid biosynthesis. Even when the meaning of these data for the competence of the oocyte to develop to the blastocyst stage remains speculative, the cumulus proteome shows clear differences between COCs with and without maturational competence. Based on these data, protein markers in the cumulus for the oocytes maturational competence can be further investigated. This has the potential to improve COC selection for a variety of assisted reproductive technologies. Additionally, the differences of protein expression between maturation conditions provide novel insights into the molecular bases of a reduced maturational competence of oocytes under *in vitro* condition. This will contribute to improve the critical step of *ex vivo* maturation in the future.

## Material and Methods

### Preparation of oocyte donor heifers and COC collection

Six healthy and cycling Brown Swiss heifers between 1 year and 9 months and 2 years and 8 months were used for this study. The study was carried out in accordance with the Swiss Animal Protection Act, permission to perform this animal experiment was issued by the Cantonal Veterinary Office of Zurich (241/2013). Cycles were synchronized with two injections of luprostiolum 11 days apart (15 mg/animal, intramuscularly; Prosolvin, Virbac, Glattbrugg, Switzerland). Oestrous was expected 2–3 days after the last PGF2α injection^73^. Follicular development and ovulation were supported using an intramuscular injection of gonadorelinum (0.25 mg/animal, intramuscularly, Fertagyl, MSD Animal Health, Lucerne, Switzerland) 48 h after the last prostaglandin injection. Successful ovulation after 24 h was controlled by ultrasonography. For the *in vivo* maturation of COCs, half of the heifers (n = 3) underwent a superovulation treatment with ovulation induction. The other three served as donors of immature COCs for *in vitro* maturation (Fig. 1). At day 9.5 of the new cycle, the dominant follicle (>1 cm) was aspirated transvaginal for synchronisation of the follicular wave, and the heifers received a progesterone-releasing intravaginal device (PRID; Prid delta, Biokema, Crissier, Switzerland).

Superovulation was initiated in three heifers at day 9.5 using eCG (2500 Units/animal, intramuscularly, Folligon, MSD Animal Health, Lucerne, Switzerland). At the same time, a mid-luteal progesterone level was ensured using a PRID in all heifers, to take advantage of the rebound effect after removal for oestrous induction. Corpora lutea regression was induced in all heifers with 15 mg luprostiolum injections (15 mg/animal, intramuscularly; Prosolvin Virbac, Glattbrugg, Switzerland) at 48 and 60 h after the PRID insertion.

In the three superovulated heifers, the PRID was removed at day 5 after eCG injection. The response to superovulation was evaluated by ultrasonography. At the same time, the cows received an intramuscular injection of 0.25 mg gonadorelinum (Fertagyl, 0.25 mg/cow intramuscular, MSD Animal Health, Lucerne, Switzerland) to induce an LH surge. The peak of the preovulatory LH surge was expected three hours after injection^74^, which was therefore defined as start for the *in vivo* maturation. The heifers were slaughtered 24 h after the last gonadorelinum injection.

The other three heifers served as oocyte donors for *in vitro* maturation. These animals were synchronised but not superovulated. Slaughtering was scheduled 6 days after the PRID insertion, without prior removal of the device. Figure 1 gives a brief schematic overview of all the performed treatments to obtain *in vivo* and *in vitro* matured oocytes. After slaughtering, ovaries were extracted from their carcasses <5 min after slaughtering. All ovaries were hold <30 min after excision in NaCl 0.9% at 35 °C containing antibiotics (0.06 g/l Penicillin; 0.1 g/l Streptomycin). Each ovary was sliced separately into a glass dish containing phosphate-buffered saline (PBS) containing heparin (2000 I.U./l) and bovine serum albumin (1 g/l). In non-superovulated heifers, dominant follicles were discarded to obtain COCs of similar stage. The medium was searched for COCs under the inverted microscope. An overview on the collected COCs and their representation in the analysis is given in Table 2.

#### *In vivo* maturation

COCs were washed in 3 subsequent 100 μl drops of holding medium (0.453 g TCM199 supplemented with 1.5 mg gentamicin sulphate; 0.66 mg sodium pyruvate; 0.03 g NaHCO_3_; 0,03 g BSA fatty acid free; dissolved in 30 ml sterile water)^75^. The transfer of COCs was conducted in a standardized volume using a Stripper micropipettor (The Stripper, MXL3–135, Origio a/s, Måløv, Denmark) adjusted to 2.5 μl. In the last drop of holding medium the oocyte was denuded from cumulus and an equal volume of 2.5 μl cumulus cells was transferred to a drop of 100 μl PBS-PVA (PBS containing 0.1 g/l polyvinyl alcohol). The cumulus was washed in three further 100 μl drops of PBS-PVA, where the cells were also transferred in a constant volume of 2.5 μl using the Stripper pipette. From the last PBS-PVA washing drop, the cumulus samples were aspirated in a volume of 2.5 μl and placed in labelled analysis tubes. The sample tubes were snap frozen in liquid nitrogen and stored until further proteomics analysis. The corresponding oocyte was fully denuded in trypsin solution (1 g/l) for further evaluation of final maturation. Extrusion of the first polar body was controlled under the inverted microscope.

#### *In vitro* maturation

Collected immature COCs were washed individually in four consecutive 100 μl drops of maturation wash medium (0.453 g TCM199 supplemented with 1.5 mg gentamicin sulphate; 0.66 mg sodium pyruvate, 0.066 g NaHCO_3_; 0.03 g BSA fatty acid free; dissolved in 30 ml sterile water) and transferred to 30 μl drops maturation medium (supplemented with 10 IU/ml equine chorionic gonadotrophin and 5 IU/ml human chorionic gonadotrophin)^75^. *In vitro* maturation was performed for 21 h in individual culture at 38.5 °C and 5% CO_2_. After maturation, COCs were transferred to 100 μl drops of holding medium and further handled as the *in vivo* matured COCs.

### Proteomic analysis

Samples from 20 single CCs (n = 5 for each group) were examined in one single proteomics analysis. A combination of sonoreactor-based (SR) cell lysis^76^ and filter-aided sample preparation (FASP)^77^ were used for protein extraction and digestion. This SR-FASP protocol was established especially for the preparation of the minute sample amounts of CCs from a single COC at the Functional Genomics Center Zurich^38^. The method described in the following paragraphs was already applied and described for equine cumulus samples^29^. As first step, samples were treated with four freeze/thaw cycles in 90% methanol. After 15 minutes the collected pellet was solved in 30 μl SDS lysis buffer (4% SDS, 100 mM Tris/HCL pH 8.2, 0.1 M dithiothreitol) and incubated at 95 °C for 5 min. Afterwards, samples were treated with High Intensity Focused Ultrasound (HIFU) for 15 min with an ultrasonic amplitude of 65% in cycle 0.5 (Sonoreactor UTR200; Hielscher Ultrasonics, Teltow, Germany). Samples were centrifuged for 10 min at 16000 *g* and protein concentration was estimated with the Qubit Protein Assay Kit (Life Technologies, Carlsbad, Ca, USA). For each sample, 10 μg of proteins were taken and used for on-filter digestion. Briefly, proteins were diluted in 200 μl of UT buffer (8 M urea in 100 mM Tris/HCL, pH 8.2), loaded on a Microcon-30kDa Centrifugal Filter Unit with Ultracel-30 membrane (Merck Millipore, Darmstadt, Germany) and centrifuged at 14,000 × *g* for 25 min at room temperature. The filter unit was washed using 200 *μ*l UT buffer and another centrifugation at 14,000 *g* for 25 min. For alkylation of reduced proteins, 100 μl iodoacetamide 0.05 M in UT buffer were added to the filter unit and incubated for 5 min. Three washing steps with 100 μl UT and two washing steps with 100 μl NaCl 0.5 M were performed. Proteins were digested overnight on the filter-unit in a wet chamber at room temperature using 120 μl of 0.05 M triethylammonium bicarbonate buffer (pH 8.5) containing trypsin (Promega, Madison, WI, USA) in a ratio 1:50 (w/w). After elution, the peptide solution was acidified using trifluoroacetic acid (TFA) to a final concentration of 0.5%. Peptides were desalted using Finisterre solid phase extraction C18 columns (Teknokroma, Barcelona, Spain), dried and resolubilized in LC-MS solution (3% acetonitrile, 0.1% formic acid) for MS analysis.

Samples were analysed with random order in one analytical run using reverse-phase LC-MS/MS on an Orbitrap Fusion mass spectrometer (Thermo Scientific, Waltham, MA, USA) operating in the data dependent acquisition (DDA) mode. The instrument was coupled to a nano-HPLC system (EASY-nLC 1000, Thermo Scientific, Waltham, MA, USA). Then, 500 ng of peptides were loaded on a self-made frit-column (75 μm × 150 mm) packed with reverse phase material (ReproSil-Pur 120 C18-AQ, 1.9 µm beads (Dr. Maisch HPLC, Ammerbuch, Germany), coupled to a fused-silica emitter (20 μm × 8 cm, tip: 10 ± 1 μm; New Objective, Woburn, MA, USA).

Solvent composition was 0.1% formic acid in water for channel A, and 0.1% formic acid in acetonitrile for channel B. Peptides were eluted at a flow rate of 300 nl/min by a gradient of 1 to 25% ACN in 50 min, 25–32% ACN in 10 min and 32–97% in 10 min. Full-scan mass spectra (300–1500 *m*/*z*) were acquired at a resolution of 120’000 at 200 *m*/*z* after accumulation to a target value of 4e5. Look mass correction was used (371,1010 and 445.12003 m/z) and the maximum cycle time between precursor mass scans was set to 3 seconds. Data-dependent MS/MS were recorded in the linear ion trap using quadrupole isolation with a window of 1.6 Da and higher-energy collisional dissociation fragmentation with 30% fragmentation energy. The ion trap was operated in rapid scan mode with a target value of 1e2 and a maximum injection time of 35 ms. Precursor signals were selected for fragmentation with a charge state from +2 to +7 and a signal intensity of at least 5e3. A dynamic exclusion list was used for 25 seconds and maximum parallelizing ion injections was activated. A pool containing 0.5 μl of each sample was analysed and used as alignment reference in data analysis.

Progenesis QI for Proteomics Software (Nonlinear Dynamics, Newcastle upon Tyne, UK) was used for label-free quantification. Automatic aligning was performed against the reference raw-file of the sample pool. Peak picking was carried out with enabled high sensitivity option and only peptide ions with the charges 2, 3 and 4 were used for analysis. Up to the top five tandem mass spectra for each detected peptide ion were exported using charge deconvolution and deisotoping option with a maximum fragment ion count of 200 peaks per MS/MS. The spectra were searched with Mascot Server v.2.5.1.3 (Matrix Science, London, UK) against the Uniprot database for Bos taurus (NCBI taxonomy ID 9913, release date 20140521) that has been concatenated with its reversed sequences for FDR estimation using the target-decoy approach. Search parameters were, a tolerance of 10 ppm for precursor ion mass and 0.5 Dalton for fragment ion tolerance, enzymatic specificity was set to trypsin allowing a maximum of two missed cleavage sites. Carbamidomethylation of cysteine was specified as a fixed modification, and oxidation of methionine, deamidation of glutamine and asparagine and protein N-terminus acetylation were selected as variable modifications. The mascot search result was loaded into Scaffold v4.1.1 (Proteome Software Inc., USA), settings for the false discovery rates were 10% on protein level and 5% on peptide level. Proteins that contained identical peptides and could, therefore, not be differentiated based on MS/MS analysis alone were grouped to satisfy the principles of parsimony. A spectrum report was exported and loaded into Progenesis QI for proteomics to link the MS1 features with peptide and protein information. Protein false discovery rate (protFDR) for the quantifiable proteins with at least two peptides was estimated to <1% using the target-decoy strategy^78^. For protein quantification, the average of the normalized abundance of the most intense 3 peptide ions of each protein group were calculated individually for each sample. This generated the normalized quantitative protein abundance^79^. Statistical testing using t-Test was performed on normalized and hyperbolic arcsine transformed protein abundances. The four experimental conditions matured *in vivo* (successfully matured and failed to mature) and matured *in vitro* (successfully matured and failed to mature) (n = 5) were compared with each other in a between subject design. Only proteins with at least two identified peptides were evaluated in the statistical analysis. Differently expressed proteins were defined with a fold change >2 along with p ≤ 0.05. The mass spectrometry proteomics data were handled using the local laboratory information management system^80^.

The mass spectrometry proteomics data have been deposited to the ProteomeXchange Consortium via the PRIDE partner repository with the dataset identifier PXD016679^81^.

Testing for overlaps in significant proteins was performed in the web-based tool InteractiVenn (http://www.interactivenn.net)^82^. String-database (http://string-db.org) was utilized for enrichment analysis of the differently expressed proteins^83^. Up- and down-regulated proteins were overlaid to all detected proteins in the experiment (2226) and analysed for overrepresentation of KEGG pathways^84^.

### Complement C3 ELISA

The secretion of complement factor C3 from COCs during maturation was tested by analysis of maturation medium using an enzyme-linked immunosorbent assay kit for complement component C3 (ELISA KIT SEA861Bo, Cloud-Clone Corp., Katy, Tx, USA). For sampling, 35 COCs were matured in a volume of 400 μl maturation medium. For analysis COCs were removed, the maturation medium was centrifuged at 1,000 × g for 15 minutes and the supernatant snap frozen until analysis. Sampling was performed after 12 h (n = 4) and 24 h (n = 10) of maturation. As negative controls, medium without cell contact and incubation (n = 3) and medium with COC contact but incubation without cells for 24 h (n = 4) were analysed. A comparison with the *in vivo* situation was performed using follicular fluid of follicles punctured on ovaries of slaughtered animals (n = 6). All samples were analyzed in duplicate on a single ELISA plate according to the manufacturer’s instruction manual.

## Supplementary information

Supplementary Information 1.

Supplementary Information 2.

Supplementary Information 3.

Supplementary Information 4.

Supplementary Information 5.
