# Supplementary material for: The bovine cumulus proteome is influenced by maturation condition and maturational competence of the oocyte
Source: Sci Rep. 2020 Jun 18;10:9880. doi: 10.1038/s41598-020-66822-z (PMC7303117; doi:10.1038/s41598-020-66822-z)
Supplement: Supplementary file 4 — Supplementary Information 4. [file 41598_2020_66822_MOESM4_ESM.docx]

**The bovine cumulus proteome is influenced by maturation condition and maturational competence of the oocyte**

Walter, J.^1*^, Monthoux, C.^1^, Fortes, C.^2^, Grossmann, J.^2^, Roschitzki, B.^2^, Meili, T.^3,5^, Riond, B.^3^, Hofmann-Lehmann, R.^3^, Naegeli, H.^4^, Bleul, U.^1^

*^1^Clinic of Reproductive Medicine, Vetsuisse Faculty, University of Zurich, Zurich, Switzerland*

*^2^Functional Genomics Centre Zurich, University and ETH Zurich, Zurich, Switzerland*

*^3^Clinical Laboratory, Department of Clinical Diagnostics and Services, Vetsuisse Faculty, University of Zurich, Zurich, Switzerland*

*^4^Institute of Pharmacology and Toxicology, Vetsuisse Faculty, University of Zurich, Zurich, Switzerland*

*^5^Center for Clinical Studies, Vetsuisse Faculty, University of Zurich, Zurich, Switzerland*

*Corresponding author: Jasmin Walter

Clinic of Reproductive Medicine, Vetsuisse Faculty

Winterthurerst. 280

CH-8057 Zurich

[jwalter@vetclinics.uzh.ch](mailto:jwalter@vetclinics.uzh.ch)
