# Supplementary material for: The bovine cumulus proteome is influenced by maturation condition and maturational competence of the oocyte
Source: Sci Rep. 2020 Jun 18;10:9880. doi: 10.1038/s41598-020-66822-z (PMC7303117; doi:10.1038/s41598-020-66822-z)
Supplement: Supplementary file 5 — Supplementary Information 5. [file 41598_2020_66822_MOESM5_ESM.docx]

**Supplemental Data:**

Supplemental Data Table 1: Complete list of significantly differentially expressed proteins between cumulus samples of successfully matured COCs matured *in vitro* or *in vivo* (“Matured”; n = 479).

Supplemental Data Table 2: Complete list of significantly differentially expressed proteins between cumulus samples of COCs matured in vivo that matured successfully or failed to mature (“In vivo”; n = 424).

Supplemental Data Table 3: Complete list of significantly differentially expressed proteins between cumulus samples of COCs that failed to mature in vitro or in vivo (“Failed to mature”; n = 175).

Supplemental Data Table 4: Complete list of significantly differentially expressed proteins between cumulus samples of COCs matured in vitro that matured successfully or failed to mature (“In vitro”; n = 20).

Supplemental Data Table 5: Protein list of the overlap analysis for the two-group analysis successfully matured in vivo against successfully matured in vitro and/or failed to mature in vivo (Figure 4).

Supplemental Data Table 6: Result of the enrichment analysis using StringDB software (www.string-db.org) for overrepresented KEGG (Kyoto Encyclopedia of Genes and Genomes) pathways (illustrated in Figure 5 & 6).
